# A Hospital-Based Cross-Sectional Study on the Histopathology of Upper Gastrointestinal Tract Endoscopic Biopsy in Dyspeptic Patients

**DOI:** 10.7759/cureus.59508

**Published:** 2024-05-02

**Authors:** Sunil Kumar Mahto, Sujit k Murmu, Arvind Kumar, Manoj K Paswan, Smita k Gupta, Venkatesh N, Tripti Ashu

**Affiliations:** 1 Pathology, Rajendra Institute of Medical Sciences, Ranchi, IND; 2 Pathology, Mahatma Gandhi Memorial Medical College, Jamshedpur, IND; 3 Community Medicine, Rajendra Institute of Medical Sciences, Ranchi, IND

**Keywords:** biopsy, git endoscopy, dyspepsia, non-neoplastic and neoplastic lesions of upper gastrointestinal tract, histopathology

## Abstract

Background

Dyspepsia is one of the most common GI complaints encountered in clinical practice. Histopathological assessment of endoscopic gastric mucosa biopsy is crucial to delineate the exact cause of dyspepsia to guide patients' management.

Objectives

The aim of this study was to determine the histopathological spectrum of upper gastrointestinal (GI) tract endoscopic biopsies and to study the age and sex distribution of the predominant upper GI lesions.

Methods

A cross-sectional study was conducted in the Department of Pathology, Rajendra Institute of Medical Sciences, Ranchi, Jharkhand, India, from January 2022 to December 2023. All endoscopic mucosal biopsies of the esophagus, stomach, and duodenum (first and second parts) lesions were examined under a microscope for histopathological findings.

Results

Out of 250 endoscopic biopsies studied, there were 76 cases of esophageal biopsies, 149 cases of gastric biopsies, and 25 cases of duodenal biopsies. The male-to-female ratio was 1.2:1. Non-neoplastic lesions were more common than neoplastic lesions. The most common lesions encountered were esophagitis in the esophagus, gastritis in the stomach, and duodenitis in the duodenum.

Conclusion

The main organic cause of dyspepsia in our setting was chronic gastritis. We conclude that endoscopy of the upper GI tract and histopathological examination help in the earlier detection of both benign and malignant lesions. This aids in better timely management of the patients and improves the overall treatment provided resulting in a better prognosis.

## Introduction

The word "dyspepsia" refers to persistent or recurrent upper abdominal pain or discomfort [1]. Dyspepsia translates to "difficult digestion" and is derived from the Greek words "dys" and "pepse" [2]. Functional dyspepsia is one of the most common functional gastrointestinal disorders and affects more than 20% of the population. The three subtypes are epigastric pain syndrome (EPS), postprandial distress syndrome (PDS), and overlapping PDS and EPS. Functional dyspepsia is diagnosed based on the Rome IV criteria. Functional dyspepsia is defined by the presence of one or more of the following symptoms: epigastric pain or burning, early satiety, and postprandial fullness in the absence of structural disease using imaging or endoscopy [3-5]. Diagnosis of functional dyspepsia requires that the symptoms be present for at least three days per week for three continuous months with an onset at least six months before the diagnosis [6].

Around 4-5% of consultations with general practitioners and 20-40% of consultations with gastroenterologists are related to dyspepsia [7]. Research from Asia shows that younger age groups have a higher prevalence of dyspepsia. According to a study conducted in urban Mumbai, India, those over 40 years of age were more likely to have dyspepsia [8]. There is a dearth of information on the clinical characteristics of dyspepsia in Ranchi, a region where smoking and tobacco use are very common. Individuals who exhibit "alarm" symptoms, younger patients who are not responding to empirical treatment, and older adults who have recently developed dyspepsia need to be investigated right away to rule out significant gastrointestinal disorders. Weight loss, anemia, vomiting, hematemesis, melena, and dyspepsia are warning signs [9].

In clinical practice, gastrointestinal tract (GIT) disorders are the most often encountered problems. They significantly increase morbidity and mortality [10]. GIT can develop a variety of lesions, including non-neoplastic illnesses. Infection, inflammation, physical and chemical injuries, vascular abnormalities, etc., are more common. In GIT, polyps can also be hyperplastic, inflammatory, adenomatous, or carcinomatous [11].

Endoscopy has limited diagnostic utility until the lesion seen during endoscopy is subjected to histopathological analysis. Diagnoses and abnormalities must be able to be identified for an endoscopy to be successful. A pathologist seldom has direct experience with the macroscopic morphology of the lesions, except malignant tumors that are excised. Correlation between the histological and endoscopic results, however, is crucial for an appropriate diagnosis. Therefore, it is critical for a pathologist to understand various endoscopic lesion patterns that could result in particular histological findings [12].

Endoscopy was first used in 1986; the upper gastrointestinal flexible fiber optic endoscope revolutionized the way in which GIT abnormalities could be diagnosed [13]. Endoscopic biopsy examination followed by histologic assessment is a convenient procedure and the current gold standard for accurate objective assessment of patients with symptoms of upper GIT. It is used not only to diagnose malignant and inflammatory lesions but also to monitor the course, extent of disease, response of the therapy, and early detection of complications. This is reflected by a rising trend in obtaining mucosal biopsies from upper GIT [14,15].

Understanding the connection between the cause of dyspepsia and the site of malignancy in various populations requires careful consideration of demographic and clinicopathological features. This crucial information was lacking in Jharkhand's population, which forms the rationale of the study. Therefore, the objective of the study is to describe the demographic and pathological presentation of cases with dyspepsia and undergone biopsy at a tertiary hospital in Ranchi.

## Materials and methods

This was a retrospective cross-sectional study carried out at Rajendra Institute of Medical Sciences (RIMS), Ranchi, Jharkhand, India. The study was approved by the Institutional Ethics Committee of RIMS Ranchi (MEMO NO-53, dated November 16, 2021). The study was conducted by collecting biopsy materials of patients enrolled through the outdoor and indoor Department of Surgery RIMS Ranchi from January 2022 to December 2023. As there was no previous study on the prevalence of dyspepsia, non-probability method of sampling was used to achieve the sample size. The sample size was determined based on the previous year's biopsy data from the institutional record. A total of 250 cases (upper gastrointestinal biopsies) were received in the histopathology laboratory, and the same was taken using consecutive sampling. Upper GIT endoscopy was conducted on all patients suffering from dyspepsia, and suspected tissue was taken out and kept inside fixative (10% formalin) followed by conventional tissue processing and embedding; 5-micron thick sections were cut, and slides were prepared. Each section was stained with hematoxylin and eosin and examined for various histopathological features. The findings were then correlated with age sex and clinical presentations.

Inclusion criteria included the following: patients aged more than 18 years who had been experiencing dyspepsia for more than three months and whose symptoms had started more than six months prior to diagnosis, and all esophageal, stomach, and duodenum (first and second parts) endoscopic mucosal biopsies.

Exclusion criteria included the following: patients less than 18 years of age, those who were not suitable for endoscopies, such as patients with shock, acute perforation, or acute myocardial infection, patients presenting with lesions in the oral cavity and pharynx and those presenting with lesions beyond the second part of the duodenum, and resection specimens.

**Figure 1:**
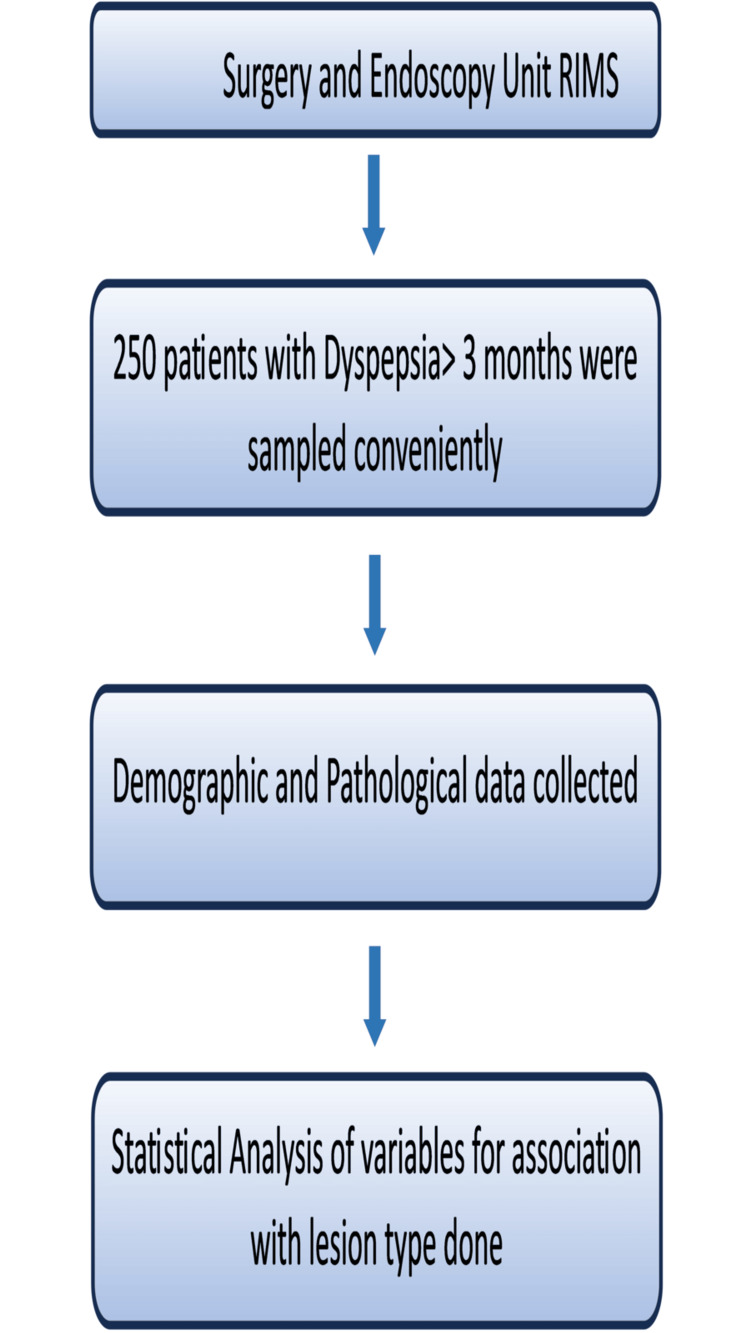
A flowchart showing the overall methodology of the study RIMS, Rajendra Institute of Medical Sciences

A pre-tested, semi-structured questionnaire was used to collect data regarding the sociodemographic profile, symptoms, signs, endoscopic results, and other data relevant for the study. We agree that as it was not a community-based study, this study holds limitations pertaining to its hospital-based and cross-sectional design. Still relating to feasibility, the study was conducted in this design. The study protocol followed the Strengthening the Reporting of Observational Studies in Epidemiology (STROBE) guidelines. The whole methodology of the study has been briefed in Figure 1.

## Results

Within the study period, a total of 250 patients had undergone endoscopic biopsy for dyspepsia, of which 137 (54.8%) were male and 113 (45.2 %) were female (Table 1). The male-to-female ratio was 1.2:1, and the mean age of the patients was 52.5 ± 13.1 years, with an age range of 24 to 77 years. In our study, we observed that males, age ≥ 50 years, people belonging to the Hindu religion, smokers, and alcoholics had undergone endoscopic biopsy followed by histopathological examination for dyspepsia than the others. The prevalence of lesion was higher at the site of the stomach, and most of them were benign lesions (Table 1).

**Table 1:** Demography and distribution of variables in the study (n=250)

Variable	Frequency (N)	Percentage (%)
Gender		
Females	113	45.2
Males	137	54.8
Age		
<50 years	96	38.4
≥50 years	154	61.6
Religion		
Hindu	176	70.4
Muslim	18	7.2
Christian	13	5.2
Sarna	43	17.2
Habit of smoking		
Non-smoker	92	36.8
Smoker	158	63.2
Habit of alcohol		
Non-alcoholic	102	40.8
Alcoholic	148	59.2
Site of lesion		
Esophagus	76	30.4
Stomach	149	59.6
Duodenum	25	10
Type of lesion		
Benign	198	79.2
Malignant	52	20.8

In our study, we found patients exhibiting multiple lesions at the same site; the separate distribution of the lesion according to the site is given in Table 2.

**Table 2:** Distribution of lesions in the study (n=250)

Lesion	Frequency(n)	Percentage (%)
Esophageal biopsy (n=76)		
Esophagitis	18	23.7
Dysplasia	20	26.3
Barett’s esophagus	31	40.8
Squamous cell carcinoma of the esophagus	7	9.2
Gastric biopsy (n=149)		
Gastric ulcer	29	19.5
Gastritis	74	49.7
Normal	8	5.4
Squamous cell carcinoma of the stomach	9	6
Adenocarcinoma of the stomach	22	14.8
Gastric lymphoma	7	4.7
Duodenal biopsy (n=25)		
Duodenal ulcer	6	24
Duodenitis	12	48
Adenocarcinoma duodenum	7	28

Statistical significance was observed in the age group of 50 years and older for the presence of malignant lesions, with the stomach being identified as a significant site for malignant lesions, both with a p-value of less than 0.05 (Table 3).

**Table 3:** Analysis of variables versus lesions (n=250)

Variable	Subgroups	Benign		Malignant		Chi-square value	P-value
		n	%	n	%		
Gender	Female	95	47.98	18	34.62	2.97	0.085
	Male	103	52.02	34	65.38		
Age	<50 years	85	42.93	11	21.15	8.256	0.004
	≥50 years	113	57.07	41	78.85		
Religion	Hindu	133	67.17	43	82.69	5.838	0.12
	Muslim	17	8.59	1	1.92		
	Christian	12	6.06	1	1.92		
	Sarna	36	18.18	7	13.46		
Smoking history	Non-smoker	68	34.34	24	46.15	2.47	0.116
	Smoker	130	65.66	28	53.85		
Alcohol intake	Non-alcoholic	76	38.38	26	50	2.301	0.129
	Alcoholic	122	61.62	26	50		
Site of lesion	Esophagus	69	34.85	7	13.46	8.984	0.011
	Stomach	111	56.06	38	73.08		
	Duodenum	18	9.09	7	13.46		

**Figure 2:**
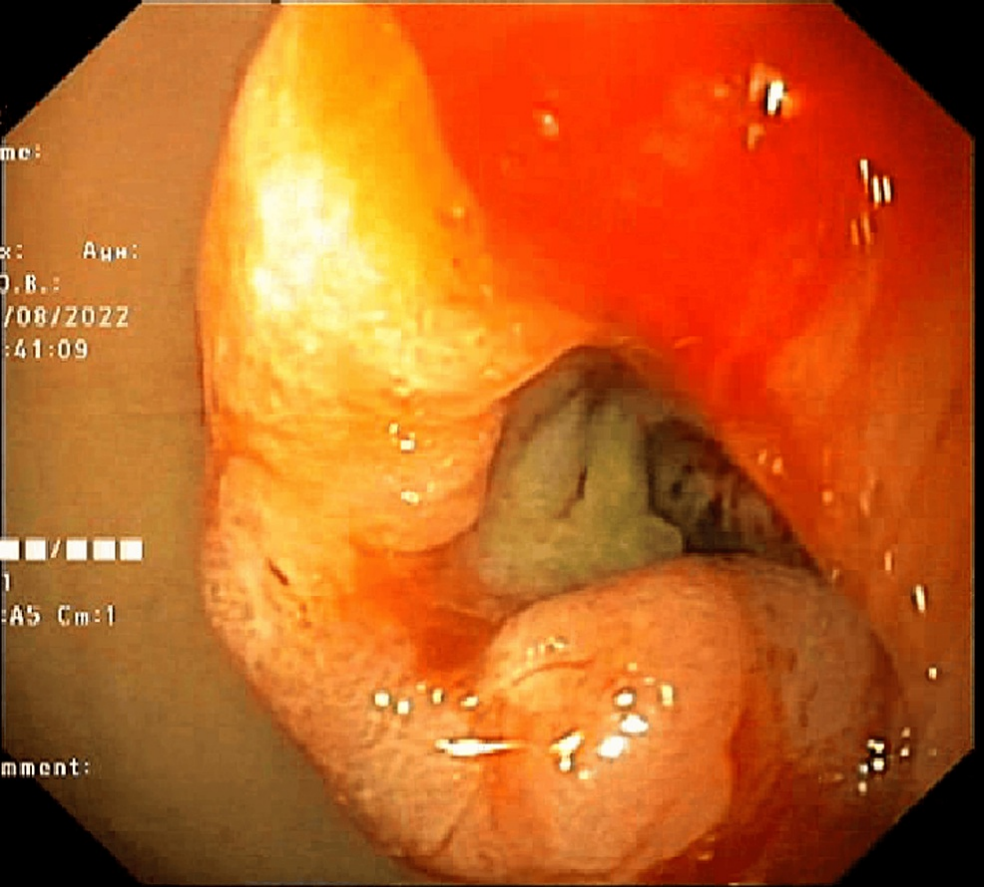
A large antral malignant ulcer

**Figure 3:**
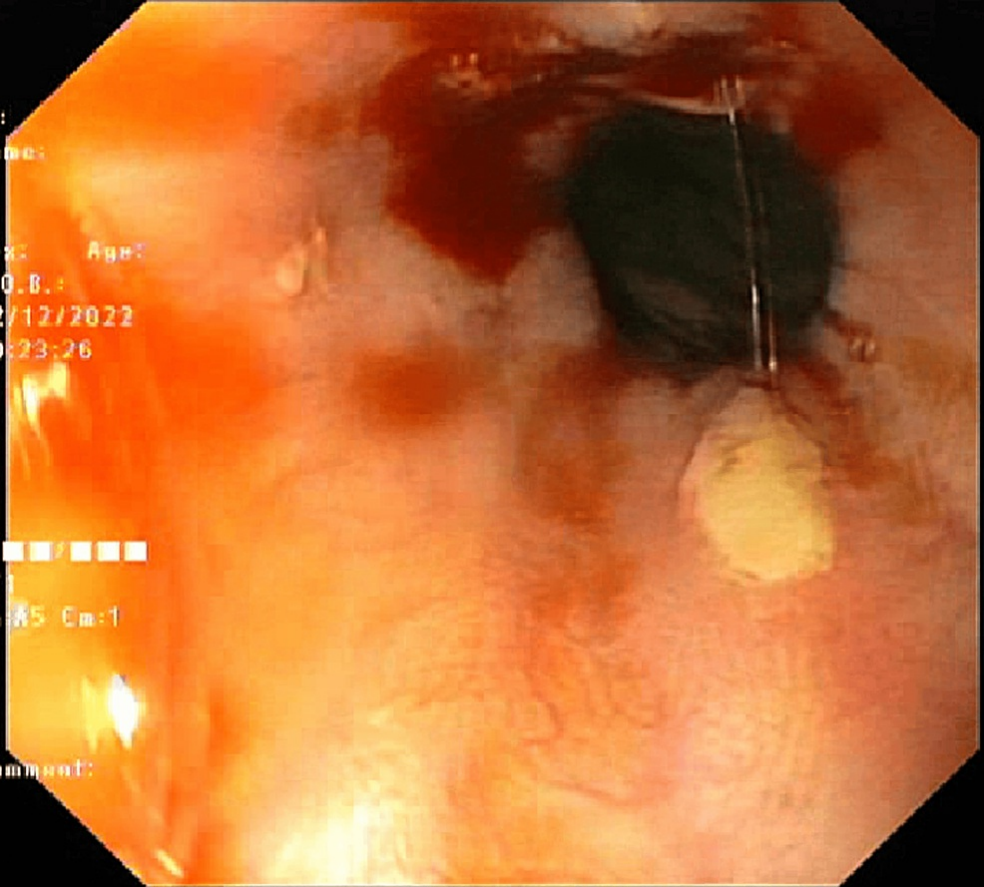
Bleeding ulcers in the esophagus

**Figure 4:**
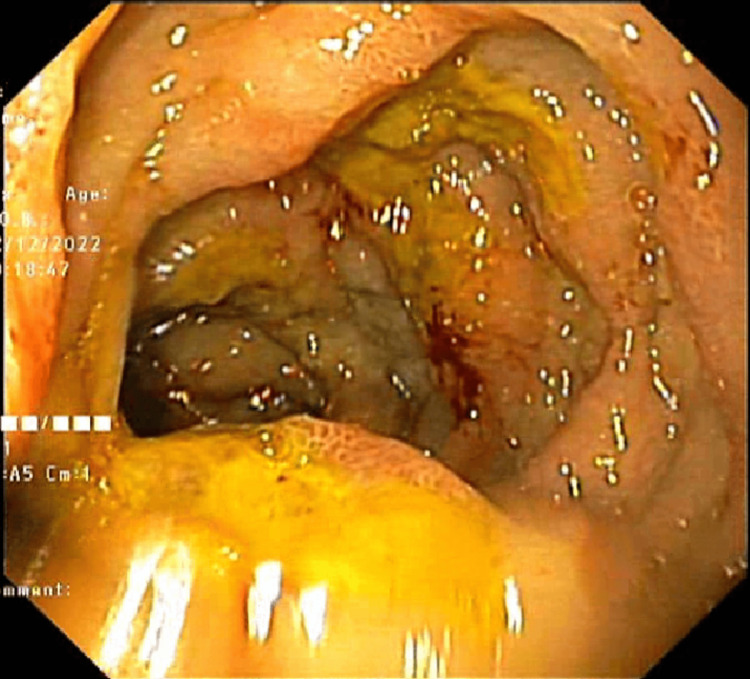
Multiple duodenal ulcers with yellow necrotic base

The endoscopic images are shown in Figures 2-4.

**Figure 5:**
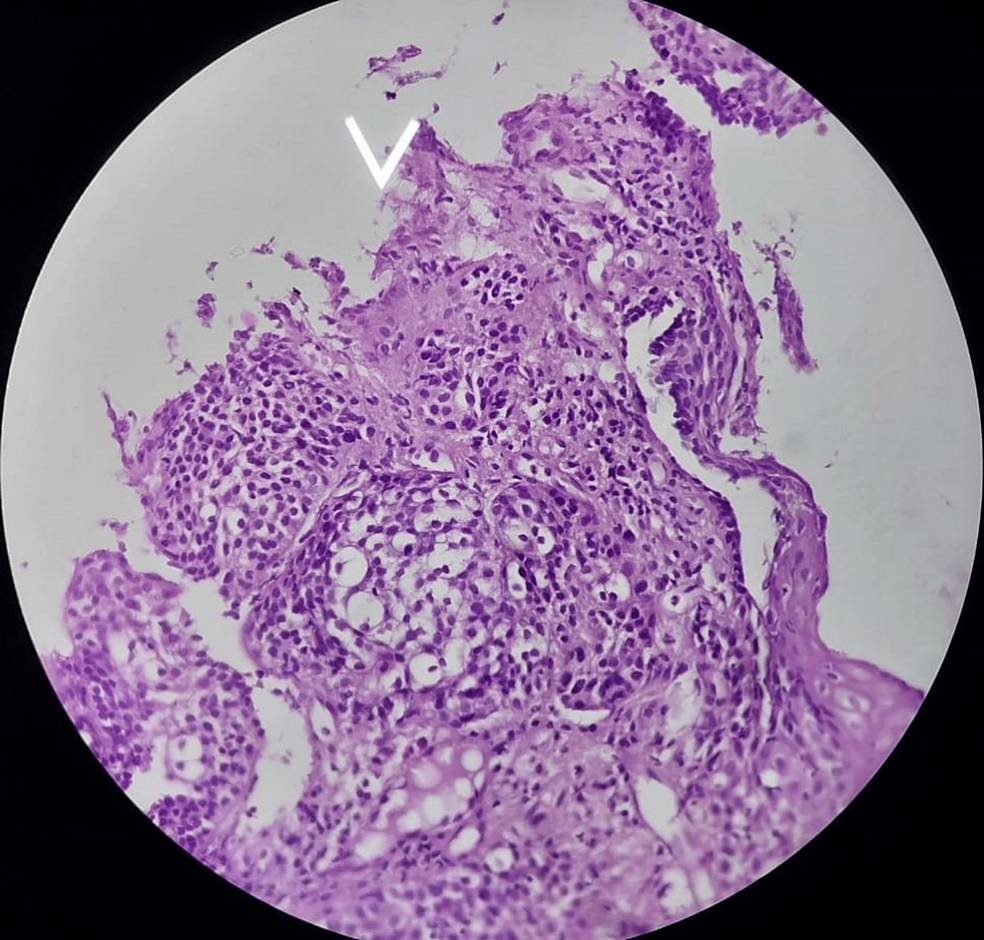
Reflux esophagitis

**Figure 6:**
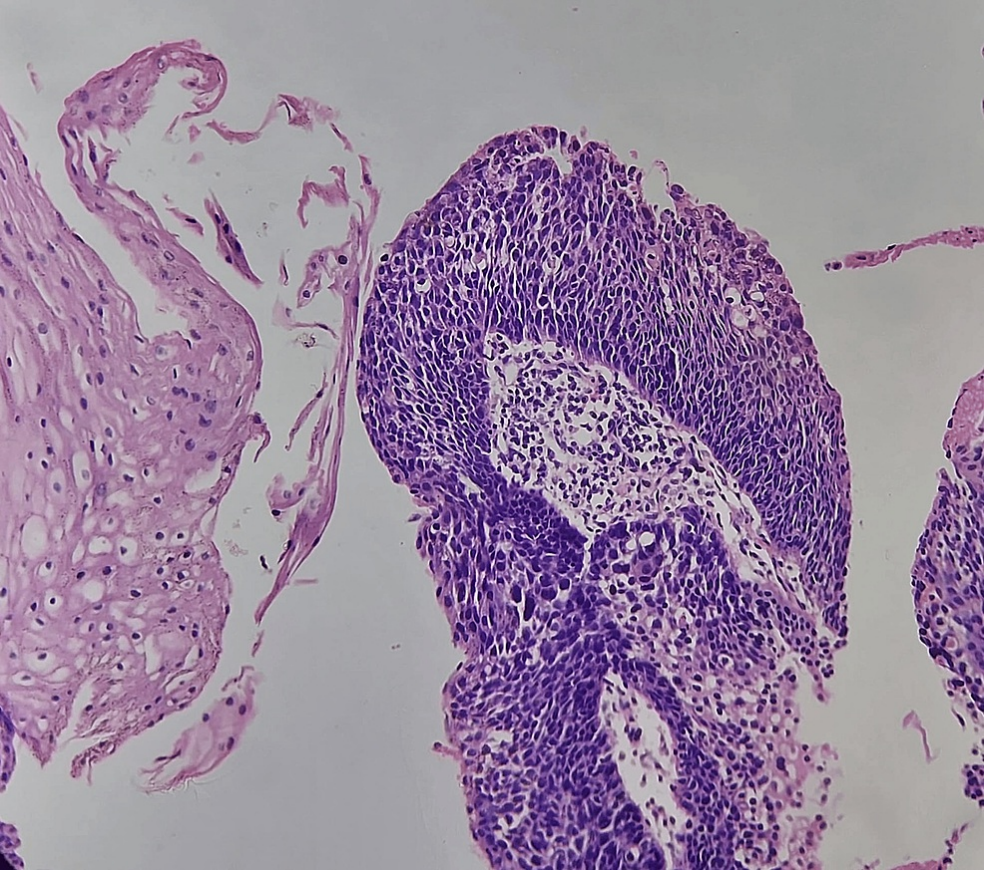
Esophageal dysplasia (40x)

**Figure 7:**
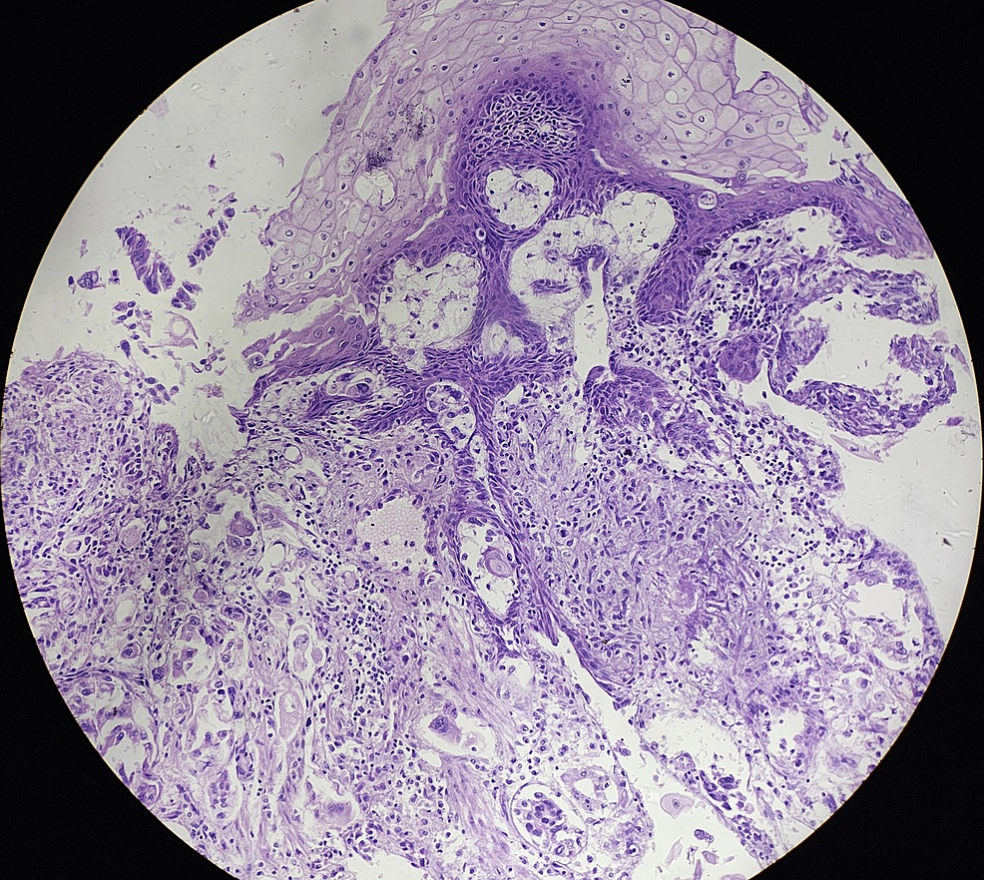
Squamous cell carcinoma of the esophagus (20x)

**Figure 8:**
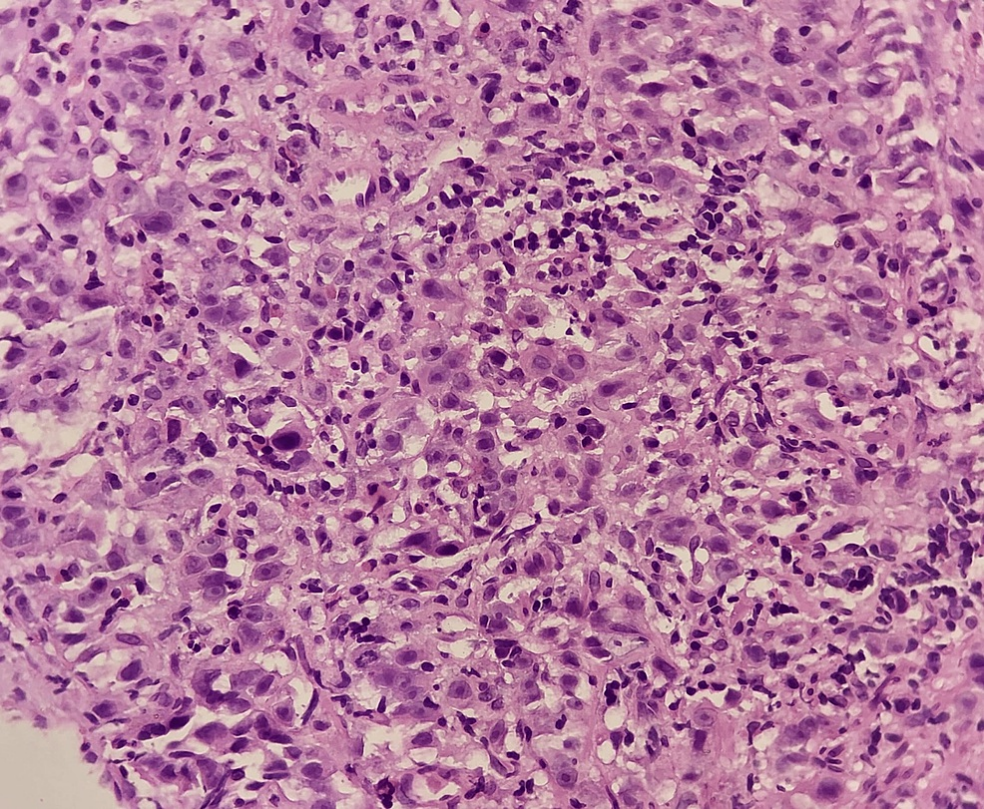
Squamous cell carcinoma of the lower end of the esophagus (40x)

**Figure 9:**
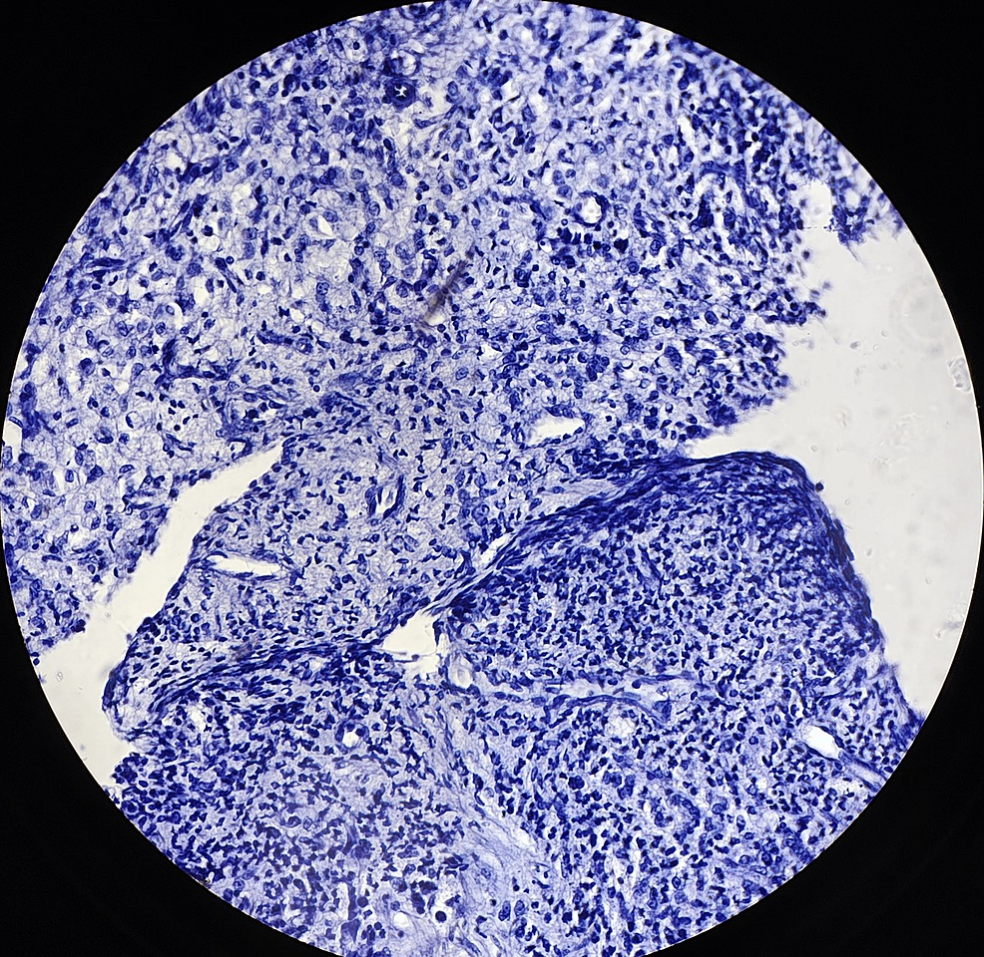
Diffuse adenocarcinoma of the stomach (40x)

The histopathological images (hematoxylin and eosin) of reflux esophagitis, esophageal dysplasia, squamous cell carcinoma esophagus (20x and 40x), and diffuse adenocarcinoma stomach showing signet ring cells are shown in Figures 5-9, respectively.

## Discussion

The current study analyzed 250 upper GIT endoscopic biopsies, comprising 76 esophageal, 149 gastric, and 25 duodenal biopsies. There were 45% female and 55% male participants in our study. The ratio of men to women was 1.2:1. This ratio favoring men may be due to the fact that men are more likely than women to be exposed to risk factors and stress. However, our data do not support a statistically significant association between gender and malignancy.

These findings were in concordance with a study conducted by Mithun et al. [15], who revealed that in his study out of 220 cases evaluated, 127 (58%) were males and 93 (42%) were females. Sharma et al. [16] also claimed in their study that out of 50 cases evaluated, 58% were males and 42 % were females.

The most common age group affected in our study was that between 50 and 60 years. This shows that elderly patients are more prone to gastrointestinal complaints because aging and the accompanying decline of various physiological processes have significant effects on the GIT. The association of age group with malignant lesions is statistically significant in our study (p<0.05).

Research by Jeshtadi et al. [17] and Florian Frochlich et al. [18] revealed similar results, reporting that patients older than 50 years comprised the majority of those who underwent endoscopic operations for upper gastrointestinal problems.

Of the participants in this study, 63% were smokers and 59% were alcoholics. This was due to the fact that there were more men than women in our sample and that no women reported a history of alcohol or tobacco use. Smoking relaxes the lower esophageal sphincter, which can exacerbate symptoms of gastroesophageal reflux. Moreover, it has been demonstrated that smoking cigarettes damages the stomach mucosa. These results were consistent with a study conducted by Hirota et al. [19], which found that 60% of the participants were alcoholics and 50% of the participants were smokers. In our research, there was no association between malignancy and drinking or smoking. In the present study, 76 (30.4%) biopsies were from the esophagus, 149 (59.6%) were from the stomach, and 25(19%) were from the duodenum. These findings were in accord with studies conducted by Farzana et al. [11], Abilash et al. [20], and Shanmugasamy et al. [21]. This may be because stomach biopsies have a bigger surface area than esophageal and duodenal samples, making them easier to access during endoscopy. In our study, the site of the stomach has a significant association with malignant lesions with p<0.05.

In contrast to the studies by Aparajita et al. [22], and Abilash et al. [20], which revealed that squamous cell carcinoma accounted for 71.4% and 42.11% of cases, respectively, malignancy was detected in only 9.2% of cases in our study. This is likely because not all dysplasia will develop into an invasive or higher-grade adenocarcinoma; in certain situations, it may even regress. If there isn't currently an invasive adenocarcinoma, even high-grade dysplasia can last for years before progressing to invasion. These factors highlight the limitations of dysplasia as a risk indicator.

In our study, out of the 25 gastric biopsies sampled, the most common lesion was gastritis, seen in 74 cases (49.7%). These findings were concordant with studies conducted by Abilash et al. [20] and Kothari et al. [23], as in both of these studies the most common lesion seen was gastritis, but discordant with that of Aparajita et al. [22], as the most lesion seen in this study was gastric adenocarcinoma. Despite careful selection of the patients having strong indications for biopsy, 5.4% of cases had normal histology.

Of the 25 duodenal samples examined in our study, 12 (48%) patients had duodenitis and 7 (28%) had adenocarcinoma of the duodenum. These results were in line with research by Kothari et al. [23], Aparajita et al. [22], and Abilash et al. [20].

Of the 76 esophageal biopsies in our investigation, seven (9.2%) cases had neoplastic (SCC) characteristics, while 69 (90.8%) cases did not have any neoplastic characteristics. Comparatively speaking, non-neoplastic lesions were more frequent. The reason for this might be that samples were taken from every patient, even those with minor stomach issues. Our findings therefore are in disagreement with those of Kishan et al.'s study [24], which found that 82% of the cases had squamous cell carcinoma identified.

Among the 149 gastric biopsies that were sampled, 31 (20.8%) cases had neoplastic lesions, while 118 (79.2%) cases did not, which suggests that in the stomach, the prevalence of non-neoplastic lesions is higher than neoplastic lesions. Because 34% of the clinical diagnoses in our sample were for gastritis, these results were in line with research by Shanmugasamy et al. [21], Abilash et al. [20], and Jonnalagadda et al. [25].

Of the duodenal cases, 18 (72%) did not have cancer. Shanmugasamy et al.'s and Ahilash et al.'s studies [20,21] yielded results similar to our study. As far as we are aware, our work provides the first study to describe the demographic, pathological, and outcome of malignancy in dyspeptic patients in Jharkhand. Given the similarities in their institutional and population characteristics, this could potentially indicate the condition of developing countries overall.

Limitations

The limitations of this study include it being a retrospective single-institution study instead of a community study, which may be more representative. Also, immunohistochemistry and molecular studies could not be conducted. Endoscopic biopsy cannot assess functional diseases. It cannot detect wall thickness and luminal diameter. It is difficult to diagnose if biopsy samples are very small. Complications of endoscopic biopsy are very rare with well-experienced endoscopic surgeons, and include perforation, laceration of major blood vessels, and mucosal bleeding.

## Conclusions

In the present study, we observed that the most common site of endoscopic biopsies was the stomach (60%, n=149). Non-neoplastic conditions predominate over neoplastic conditions. In the stomach, the most common non-neoplastic lesion is chronic gastritis, which presents as patches of erythema, and adenocarcinoma presenting as ulcerative growth is more prevalent among the neoplastic lesions. Squamous cell carcinoma presenting as growth and chronic duodenitis presenting as multiple nodules are common lesions of the esophagus and duodenum, respectively. Further large-scale studies are recommended to search for environmental causes that give rise to upper gastrointestinal pathologies. Further studies incorporating genetic and molecular testing are recommended to better understand the molecular mechanisms of upper GI malignancies. Endoscopic procedure in adjunction with histological interpretation remains the mainstay of diagnostic modality in cases of upper GIT lesions.
